# Connecting urban green and blue spaces with children’ health: a bibliometric analysis in CiteSpace and VOSviewer

**DOI:** 10.3389/fpsyg.2025.1560467

**Published:** 2025-05-09

**Authors:** Yutong Kang, Zhengbing Guo

**Affiliations:** ^1^Xi'an Innovation College of Yan'an University, Xi'an, China; ^2^School of Public Policy and Administration, Xi’an Jiaotong University, Xi'an, China

**Keywords:** urban green space, urban blue space, children’s health, CiteSpace, VOSviewer

## Abstract

**Introduction:**

Rapid urbanization has increasingly restricted children’s access to natural environments, raising concerns about potential consequences for their physical, mental, and social well-being. Urban green and blue spaces are known to offer significant health benefits, including physical activity promotion, psychological restoration, and social development.

**Methods:**

This study conducted a comprehensive bibliometric analysis to examine the relationship between urban green/blue spaces and children’s health. A total of 575 relevant publications from 1981 to 2024 were retrieved from the Web of Science database. CiteSpace and VOSviewer were used for keyword co-occurrence analysis, co-citation mapping, and burst detection to visualize research trends and thematic evolution.

**Results:**

Three major research phases were identified: (1) early focus on physical health outcomes, (2) a shift toward urban environmental contexts, and (3) emerging emphasis on sustainability, environmental quality, and walkability. While green spaces have been extensively studied, blue spaces remain underrepresented, especially in terms of their synergistic benefits when combined with green spaces. Key research themes include mental and physical health impacts, social skills development, and the educational functions of nature exposure.

**Discussion:**

This study reveals the interdisciplinary and collaborative nature of current research and emphasizes the importance of ensuring equitable access to high-quality natural environments in urban areas. The findings offer practical implications for urban planners and policymakers and establish a research foundation for promoting sustainable urban development that supports children’s health and well-being.

## 1 Introduction

The unprecedented pace of urbanization has significantly transformed living environments, particularly for children (Aliyu and Amadu, 2017). Urban landscapes, dominated by concrete and asphalt, have limited opportunities for children to connect with nature (Hand et al., 2018), which has raised serious concerns about its impact on their physical and psychological health. Over recent decades, the importance of nature exposure during childhood has been increasingly recognized. Childhood is a formative period that plays a critical role in emotional, cognitive, and social development. Studies highlight that limited opportunities for exposure to natural environments contribute to higher levels of stress, anxiety, and depression in children (Liu and Green, 2023).

Green and blue spaces, encompassing parks, gardens, rivers, lakes, and coastal areas, have emerged as valuable urban resources for promoting public health. Their benefits for children include facilitating physical activity, fostering social interactions, and enhancing psychological well-being through exposure to restorative environments (Wray et al., 2020; Hernández-Torrano et al., 2020). These spaces not only reduce environmental stressors, such as noise and air pollution, but also serve as catalysts for creativity, exploration, and play. These activities are critical for childhood development. As cities continue to expand, ensuring equitable access to such spaces has become a pressing concern for policymakers and urban planners (Zong et al., 2024).

The potential of green and blue spaces to mitigate urban living’s adverse effects has been widely acknowledged, but the evidence regarding their specific benefits for children remains fragmented. While studies on adults have consistently shown associations between access to natural spaces and reduced stress levels, improved mood, and enhanced cognitive performance (Chan et al., 2023), research on children is often underrepresented or methodologically inconsistent. Most studies are cross-sectional in design, limiting the ability to draw causal inferences, and focus primarily on green spaces near residences, overlooking the importance of school environments and community spaces.

Moreover, blue spaces, such as lakes and rivers, are underexplored compared to green spaces. While green spaces have been widely associated with improved attention, reduced behavioral issues, and enhanced mental well-being in children, the unique contributions of blue spaces remain poorly understood. Additionally, few studies consider the combined effects of green and blue spaces, which might have synergistic benefits for children’s health and development.

The restorative benefits of green and blue spaces can be understood through environmental psychology theories, particularly the Stress Recovery Theory (SRT; Ulrich et al., 1991) and the Attention Restoration Theory (ART; Tennessen and Cimprich, 1995). SRT posits that natural environments promote physiological recovery from stress by reducing blood pressure and enhancing emotional well-being. ART, on the other hand, highlights nature’s role in restoring depleted attention resources, allowing individuals to recover from mental fatigue and regain focus. For children, these theories are particularly relevant, as their developmental stages involve heightened sensitivity to stressors and a greater need for attentional restoration through engaging, yet calming, environments. Our study conducts a comprehensive and systematic bibliometric analysis using CiteSpace and VOSviewer. It focuses on the impact of urban green and blue spaces on children’s health. The analysis examines the current state, trends, and hotspots of research from 1981 to 2024. The objectives of our study are as follows: (1) to provide an overview of the field from the perspectives of time, journals, research areas, international collaborations, and author networks; (2) to reveal the current state and research hotspots of the field through keyword clustering analysis; (3) to explore research frontiers based on keyword burst analysis; (4) to discuss milestone studies in the field through co-citation analysis. The findings of our study offer researchers and policymakers an accurate and systematic description of the literature on the impact of urban green and blue spaces on children’s health. These insights aim to support sustainable urban development and promote children’s health and well-being. By understanding these trends, new scholars and researchers can identify future research directions and questions through interdisciplinary collaboration.

## 2 Materials and methods

### 2.1 Data sources

The data for the bibliometric analysis was sourced from the Web of Science (WOS) Core Collection, a comprehensive and widely recognized database for high-quality academic research. WOS Core Collection was chosen for its extensive coverage of peer-reviewed journals across various disciplines, including environmental science, public health, and urban studies, which are central to our study. It offers reliable citation data and advanced search functionalities, enabling precise retrieval of relevant publications. Furthermore, WOS ensures the inclusion of only high-impact and rigorously reviewed content, making it an ideal resource for conducting bibliometric studies that require robust and credible data.

In our study, the search keywords were “urban green space,” “urban blue space,” “health,” and “children” with the specific search keywords detailed in Table 1. The search was restricted to articles and reviews written in English, and the search period extended up to November 4, 2024. A total of 1,219 search results were initially retrieved. After reviewing the titles, abstracts, and keywords, documents that merely mentioned urban green space and children’s health as a contextual background, without specifically addressing the impacts of urban blue and green spaces on children’s health in the results section, were excluded. When necessary, full texts were reviewed to confirm the inclusion criteria. Consequently, irrelevant articles lacking direct relevance to the research objective were removed, resulting in a final dataset of 575 documents selected for subsequent analysis. This selection approach ensured that the included articles provided empirical or substantial discussion of the relationship between urban blue-green spaces and children’s health outcomes. The selected literature data was exported in “.txt” format for further processing.

**Table 1:** The searching keywords on urban green and blue spaces with children’ health.

Topic	Keywords
Urban green space	TS = (“urban green space”) OR TS = (“green space”) OR TS = (“urban vegetation”) OR TS = (“urban landscape”) OR TS = (“greenness”) OR TS = (“open space”) OR TS = (“parks”) OR TS = (“gardens”) OR TS = (“yards”) OR TS = (“greenways”) OR TS = (“plaza”) OR TS = (“urban nature”) OR TS = (“green infrastructure”)
Blue green space	TS = (river) OR TS = (lake) OR TS = (“aquatic environments”) OR TS = (riparian) OR TS = (“Blue space”) OR TS = (“Water-based settings”) OR TS = (“Coastal environments”) OR TS = (“Aquatic environments”) OR TS = (“Marine environments”) OR TS = (“Bodies of water”) OR TS = (“Waterfront areas”) OR TS = (“Riparian zones”) OR TS = (“Lakeshores”) OR TS = (“Riverbanks”) OR TS = (“Ocean views”) OR TS = (“Sea views”) OR TS = (“Water features”)
Health	TS = (Well-being) OR TS = (Anxiety) OR TS = (Depression) OR TS = (Stress) OR TS = (health)
Child	TI = (Child) OR TI = (Children) OR TI = (Adolescent) OR TI = (Youth)

### 2.2 Methodology

Bibliometric analysis is a quantitative research method used to evaluate and analyze academic literature within a specific field, providing insights into research trends, impact, and structure (Kim et al., 2016; Chen, 2006; Chen, 2017). This method is widely applied across disciplines to assess research contributions. In our study, bibliometric analysis is particularly valuable for systematically exploring the intersection of green and blue spaces, health, and children, offering a comprehensive understanding of the research landscape.

In our study, bibliometric analysis was conducted using two widely recognized software, CiteSpace and VOSviewer, to analyze the data systematically. Each tool was used for specific analytical purposes, leveraging their strengths in bibliometric visualization and analysis.

First, VOSviewer was used for country co-occurrence analysis and author co-occurrence analysis, aiming to explore the structure and dynamics of scientific collaboration networks. Additionally, VOSviewer’s keyword clustering analysis was used to identify research themes and hotspots, enabling a comprehensive understanding of the focal areas within the domain.

Second, CiteSpace was used to explore the evolution of research trends and frontiers. The keyword burst detection in CiteSpace was used to highlight emerging trends and research frontiers. This helped identify the distinct stages of development within the field. Furthermore, the co-citation analysis of references of CiteSpace was used to identify milestone papers that have significantly influenced the development of the field. This analysis provided a historical perspective, highlighting foundational works and key contributions that shaped the research landscape.

## 3 Results

### 3.1 Research trend analysis

#### 3.1.1 Annual publication

**Figure 1:**
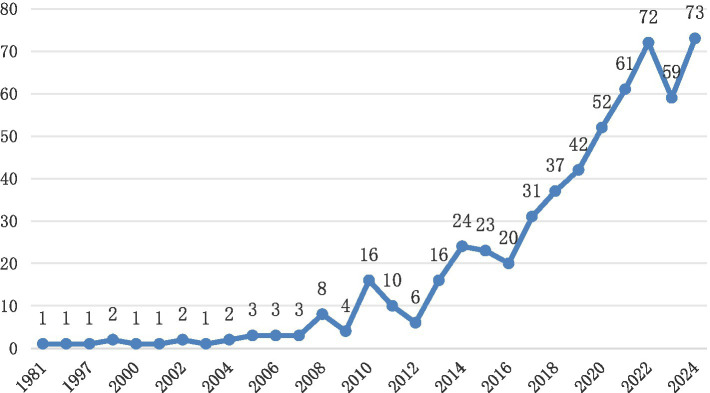
Line chart of publication trend from 1981 to 2024 (number of articles per year).

To gain a comprehensive understanding of the impact of urban blue and green spaces on children’s health, we analyzed and compiled the annual publication trends from 1981 to 2020 (Figure 1).

The publication trend of papers sourced from the Web of Science database, can be divided into three distinct phases. From 1981 to 2010, the Emerging Phase, the annual number of publications remained minimal, with only 1–3 papers per year, reflecting the infancy of the topic and limited academic attention. Between 2011 and 2015, the Growth Phase, there was a gradual increase in publications, with annual numbers rising to 6–16, indicating an initial recognition of the importance of this research area. From 2016 to 2024, the Rapid Expansion Phase, the field experienced exponential growth, with annual publications surging from 20 in 2016 to 73 in 2024. Notable milestones include a sharp rise from 31 in 2018 to 61 in 2020, and a peak of 73 publications in 2024. This trend highlights the evolution of the field from an underexplored topic to a rapidly expanding research focus.

#### 3.1.2 Journal distribution

The Table 2 identifies the journals publishing articles on the impact of urban blue and green spaces on children’s health. The International Journal of Environmental Research and Public Health leads with 57 publications, followed by Environmental Research with 35. Health & Place ranks third with 22 publications. Journals such as Preventive Medicine (18 articles) and Urban Forestry & Urban Greening (17 articles) focus on proactive health measures and the role of urban greenery. Other notable contributors include Environment International and Landscape and Urban Planning, each with 16 articles, highlighting environmental health and sustainable urban design. This distribution underscores the interdisciplinary appeal of the topic, bridging public health, urban planning, and environmental cience.

**Table 2:** The top 10 journals in number of publications on urban green and blue spaces with children’ health.

No.	Journal	Freq.
1	INTERNATIONAL JOURNAL OF ENVIRONMENTAL RESEARCH AND PUBLIC HEALTH	57
2	ENVIRONMENTAL RESEARCH	35
3	HEALTH PLACE	22
4	PREVENTIVE MEDICINE	18
5	URBAN FORESTRY URBAN GREENING	17
6	ENVIRONMENT INTERNATIONAL	16
7	LANDSCAPE AND URBAN PLANNING	16
8	JOURNAL OF PHYSICAL ACTIVITY HEALTH	14
9	SCIENCE OF THE TOTAL ENVIRONMENT	13
10	BMC PUBLIC HEALTH	12

#### 3.1.3 Research field distribution

The research on urban blue and green spaces and their impact on health spans multiple disciplines, reflecting its interdisciplinary nature (Table 3). Leading the field is Public Environmental Occupational Health, with 268 publications, followed closely by Environmental Sciences Ecology (253), emphasizing health and ecological dimensions. Other key directions include Urban Studies (46) and Geography (36), which explore spatial and urban planning aspects, and General Internal Medicine (34) and Pediatrics (25), focusing on clinical and child health impacts. Behavioral and social sciences, such as Psychology (32) and Public Administration (26), address mental health and policy implications, while interdisciplinary fields like Science and Technology (23) and Nutrition and Dietetics (22) link environmental influences to broader lifestyle factors. Together, these research directions align with the focus of top publishing journals, highlighting the integration of health, environmental sciences, and social sciences in studying the benefits of urban blue and green spaces.

**Table 3:** The top 10 research areas in number of publications on urban green and blue spaces with children’ health.

No.	Discipline	Freq.	No.	Discipline	Freq.
1	Public Environmental Occupational Health	268	6	Psychology	32
2	Environmental Sciences Ecology	253	7	Public Administration	26
3	Urban Studies	46	8	Pediatrics	25
4	Geography	36	9	Science Technology Other Topics	23
5	General Internal Medicine	34	10	Nutrition Dietetics	22

### 3.2 Analysis of research cooperation network

#### 3.2.1 Analysis of cooperation between countries

**Figure 2:**
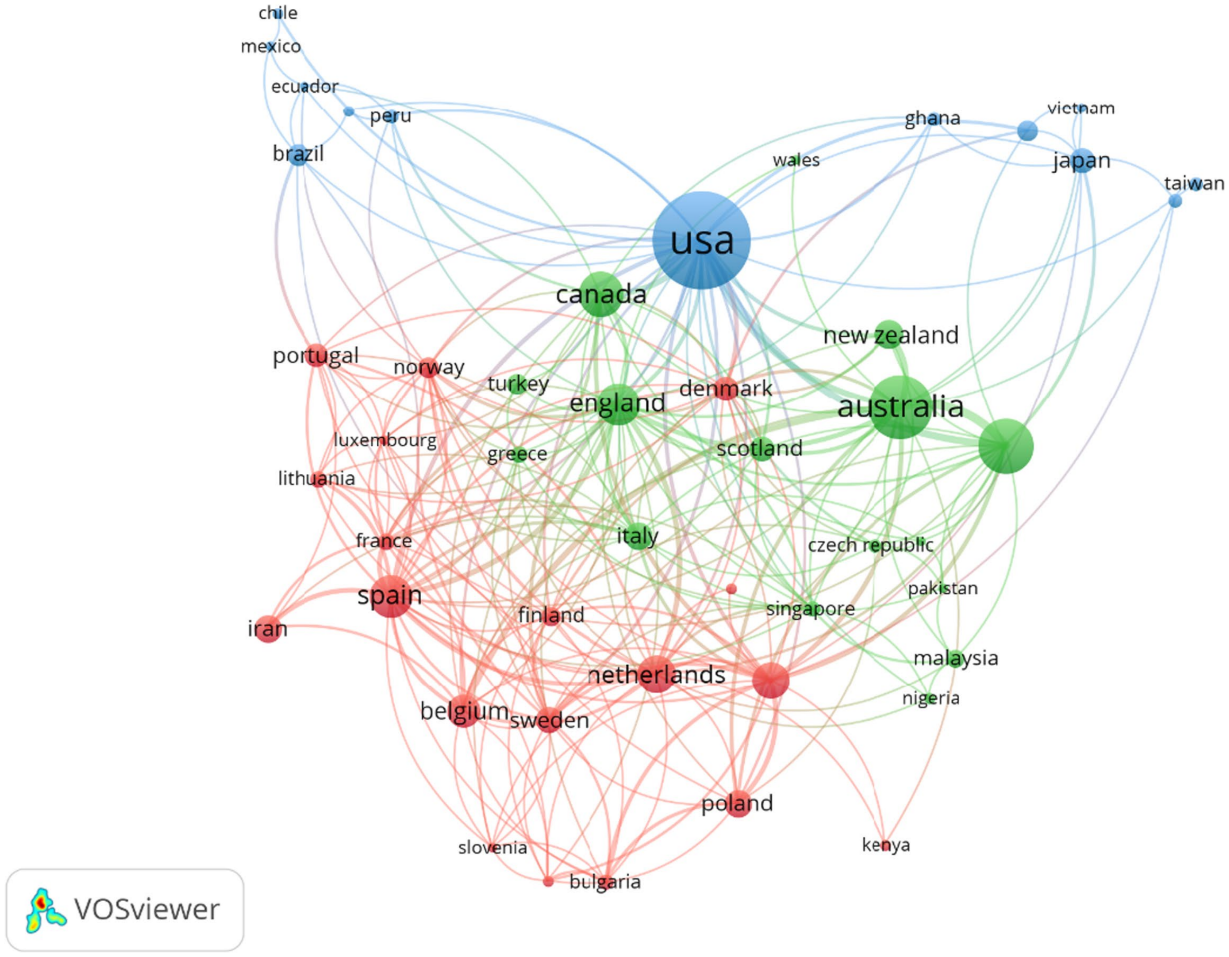
Cooperation map of countries.

The distribution (Table 4) of research on urban blue and green spaces reveals significant global participation, with the United States leading in publication frequency (200), followed by Australia (90), China (66), Canada (44), and England (40). Figure 2 presents the cooperation of countries contributing to research on the impact of urban blue and green spaces on children’s health. In this visualization, each node represents a country, and node size indicates the volume of publications from that country. Lines connecting nodes illustrate collaboration between countries, with thicker lines indicating stronger collaborative ties. Different colors distinguish academic clusters or communities that frequently collaborate. The blue cluster is centered on the United States, representing strong transcontinental collaborations, particularly with East Asia countries. The green cluster, led by Australia and New Zealand, highlights regional collaboration within Oceania and connections to Asian countries like Singapore. The red cluster, comprising Spain, Belgium, and the Netherlands, emphasizes close cooperation within Western Europe. These clusters illustrate the global and interconnected nature of this research field, with the USA, Australia, and European countries serving as key hubs of academic activity.

**Table 4:** The top 10 countries in number of publications on urban green and blue spaces with children’ health.

No.	Country	Freq.	No.	Country	Freq.
1	USA	200	6	SPAIN	40
2	AUSTRALIA	90	7	NETHERLANDS	31
3	PEOPLES R CHINA	66	8	GERMANY	29
4	CANADA	44	9	BELGIUM	23
5	ENGLAND	40	10	NEW ZEALAND	18

#### 3.2.2 Analysis of cooperation between authors

**Figure 3:**
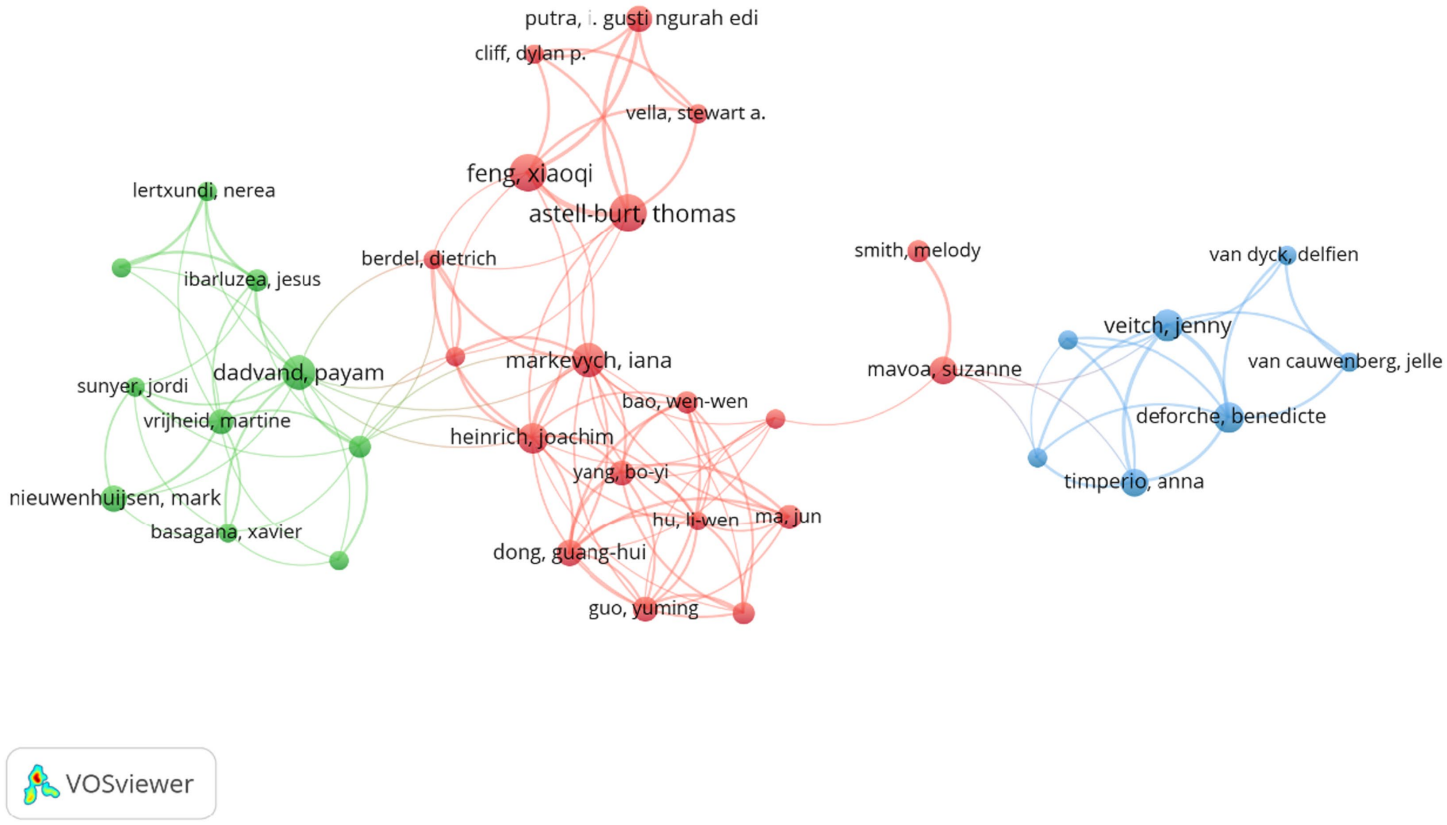
Cooperation map of authors.

The distribution of authors contributing to research on urban blue and green spaces highlights several key contributors (Table 5). Feng Xiaoqi and Astell-Burt Thomas lead with 15 publications each, followed by Markevych Iana (11), Veitch Jenny, and Dadvand Payam (10 each). Other notable contributors include Deforche Benedicte, Heinrich Joachim, and Timperio Anna (9 each). Figure 3 presents a cooperation map of authors. Each node corresponds to an author, with larger nodes representing authors with higher publication counts. The connecting lines between authors indicate co-authorship relationships, where thicker lines reflect stronger collaboration intensity. Three distinct clusters emerge, each highlighted by a different color, representing separate academic communities or research groups. The red cluster, led by Feng Xiaoqi and Astell-Burt Thomas, focuses on physical health and urban environments. The green cluster, including Dadvand Payam, emphasizes behavioral problems in children and greenspace. The blue cluster, led by Veitch Jenny and Deforche Benedicte, centers on children’s use of urban green space.

**Table 5:** The top 10 authors in number of publications on urban green and blue spaces with children’ health.

No.	Author	Freq.	No.	Author	Freq.
1	Feng, Xiaoqi	15	6	Deforchere, Benedicte	9
2	Astell-Burt, Thomas	15	7	Heinrich, Joachim	9
3	Markevych, Iana	11	8	Timperio, Anna F	9
4	Veitch, Jenny	10	9	Mavoa, Suzanne	8
5	Dadvand, Payam	10	10	Bijens, Esmee M.	7

### 3.3 Analysis of research hotspots

**Figure 4:**
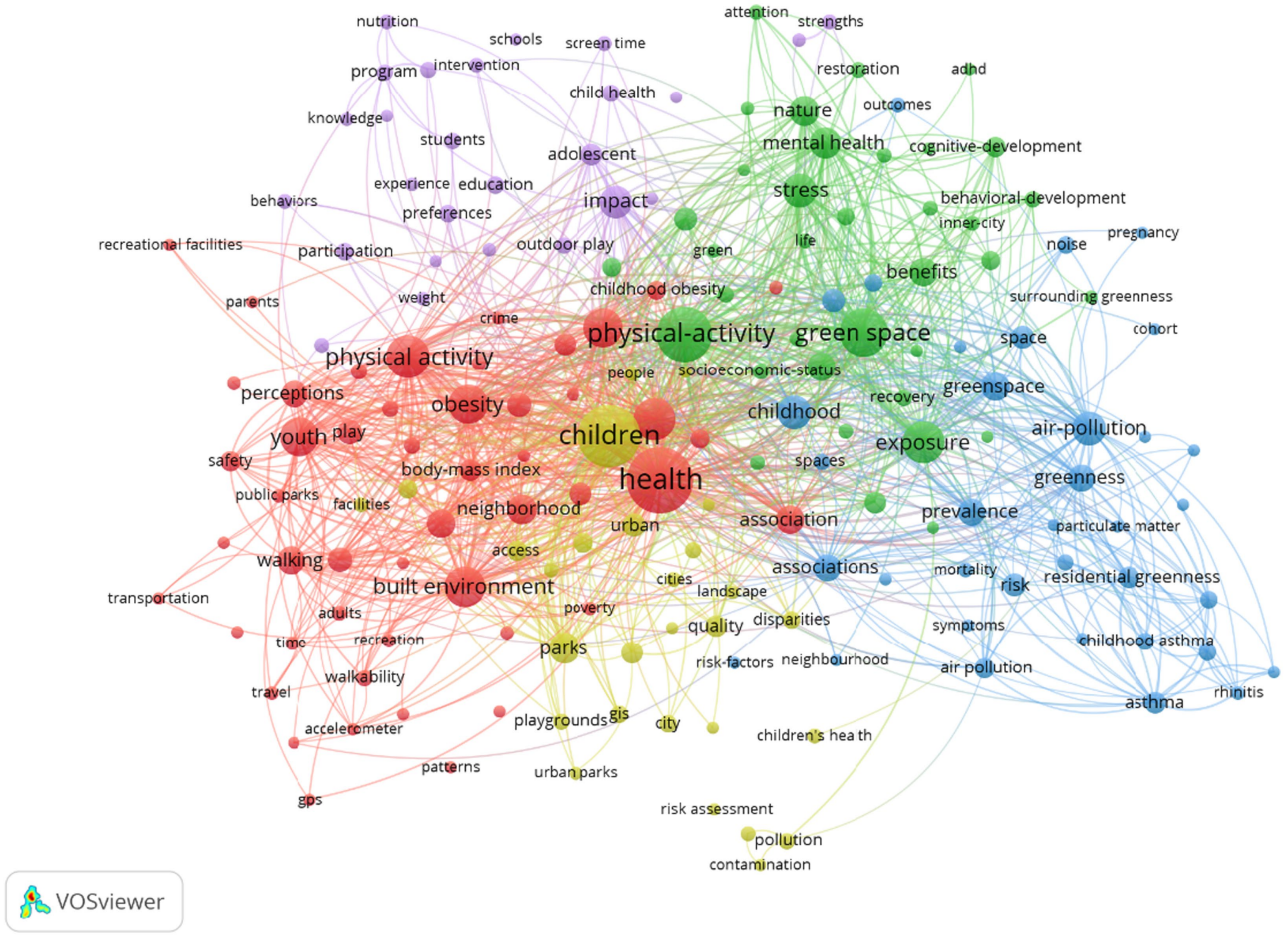
Keyword clustering analysis network.

Figure 4 is the keyword clustering analysis network in the field of research on urban blue and green spaces and children’s health. Each node represents a keyword, and the size of the node reflects the frequency of that keyword’s appearance across the literature. Links between nodes indicate co-occurrence relationships, with thicker links representing stronger associations. Different colors indicate distinct thematic clusters, revealing the main research themes in this field. The primary keywords within each thematic cluster are presented in Table 6. Five distinct clusters encapsulate the multifaceted impact of urban blue and green spaces on children’s health. The blue cluster highlights the threats posed by blue and green spaces to children’s health, while the yellow cluster focuses on the role of blue and green spaces in influencing children’s education and cognitive development. The purple cluster emphasizes the impact of blue and green spaces on children’s social skills, and the green cluster explores their influence on children’s mental health. Lastly, the red cluster centers on the relationship between blue and green spaces and children’s physical health.

**Table 6:** Keyword clustering.

Red cluster	Green cluster	Blue cluster	Yellow cluster	Purple cluster
hysical activity	mental health	air pollution	parks	students
obesity	nature	particulate matter	playgrounds	knowledge
play	benefits	prevalence	urban parks	intervention
walking	stress	asthma	quality	screen time
neighborhood	recovery	rhinitis	disparities	behaviors
recreation	restoration	contamination	city	outdoor play
facilities	exposure	mortality	neighborhood	preferences
body-mass index	cognitive development	childhood asthma	access	participation

#### 3.3.1 Threats to children’s health from blue and green spaces

Urban blue and green spaces are often hailed as vital components for promoting public health, particularly for vulnerable groups such as children. However, evidence suggests that these spaces may inadvertently become sources of significant health threats due to environmental contamination. For instance, pollutants such as heavy metals and pesticides, commonly found in water sources within urban green spaces, pose serious health risks (Alves et al., 2019). Studies have demonstrated that heavy metals like arsenic, cadmium, and chromium in Tunisian water bodies (Troudi et al., 2022) and pesticide residues in the aquifers of Mexico’s Yucatan Peninsula significantly elevate the risk of carcinogenic among children (Perera-Rios et al., 2022). These findings highlight the dual nature of urban blue and green spaces, which, while theoretically beneficial, may exacerbate health inequities when pollution persists.

Moreover, urban soils and recreational areas within green spaces often serve as reservoirs for hazardous substances, further complicating their threat in public health. Elevated lead concentrations in soils, as observed in Brooklyn, New York, and Seoul, South Korea, not only contribute to heightened blood lead levels in children but also increase their susceptibility to neurodevelopmental impairments and behavioral disorders (Byers et al., 2020). The presence of polycyclic aromatic hydrocarbons (PAHs) in playgrounds and recycled rubber surfaces further exacerbates cancer risks, raising concerns about the safety of these widely used recreational environments (Tarafdar et al., 2020). Such risks are magnified in socioeconomically disadvantaged communities, where children are more likely to encounter these exposures due to systemic inequalities in urban planning and environmental regulation (Pavilonis et al., 2022).

#### 3.3.2 The role of blue and green spaces in education and cognitive development

Urban blue and green spaces play a vital role in fostering children’s cognitive and education. Research demonstrates that early exposure to green spaces is strongly associated with enhanced cognitive functions, including improved attention, working memory, and psychomotor skills, as well as better socioemotional outcomes (Mygind et al., 2021). School gardens further stimulate curiosity, autonomy, and a deeper connection with nature, fostering long-term environmental engagement (Murray et al., 2023). However, access to these benefits is often constrained by socioeconomic inequalities, uneven distribution of green spaces, and parental concerns about safety and environmental quality (Robinson et al., 2022). Additionally, urbanization and increased screen time have exacerbated children’s disconnect from nature, reducing opportunities for exploration and developmental enrichment. To maximize the developmental potential of blue and green spaces, urban planning must prioritize safety, inclusivity, and ecological diversity, while incorporating innovative educational programs that reconnect children with nature, ultimately supporting their holistic growth.

#### 3.3.3 Impact of blue and green spaces on children’s social skills

Urban blue and green spaces play a crucial role in fostering children’s social skills by promoting interaction, collaboration, and a sense of community. Time spent in these spaces allows for unstructured play, which has been shown to enhance cooperation, communication, and trust among peers. For instance, research from Iceland highlights that children who spend more time outdoors report stronger friendships and better social integration (Thorsteinsson et al., 2023). Moreover, natural settings can mitigate the effects of social exclusion by fostering inclusivity and enhancing prosocial behaviors such as empathy and collaboration, particularly in children who have experienced rejection or peer isolation (Chen et al., 2023). Nature-based educational programs and outdoor activities, such as school gardens, further encourage teamwork and shared responsibilities, helping children develop leadership skills and improve group cohesion (Mann et al., 2022). However, challenges such as socioeconomic disparities can limit access to high-quality green spaces, restricting opportunities for social interaction and play, especially for children from disadvantaged backgrounds. Additionally, overly regulated or commercialized green spaces may constrain children’s ability to engage freely, reducing the potential for creative and spontaneous social interactions. Strategies such as integrating green spaces into school curriculums, encouraging community-led maintenance, and creating child-friendly environments can enhance their social utility (Perez-Silva et al., 2023).

#### 3.3.4 Influence of blue and green spaces on children’s mental health

Urban blue and green spaces play a significant role in influencing children’s mental health, offering benefits that enhance happiness and well-being while also mitigating stress and symptoms of depression (Li et al., 2025). Research highlights that exposure to green spaces improves emotional regulation, reduces behavioral difficulties, and promotes prosocial behaviors among children (Caryl et al., 2024). These spaces act as restorative environments, providing opportunities for relaxation, stress relief, and emotional recovery through both passive and active interactions with nature (Zhou et al., 2024). Green spaces near schools have been shown to reduce depressive symptoms in adolescents, especially when combined with limited recreational screen time (Liu et al., 2024). Additionally, coastal environments uniquely contribute to mental health by fostering a sense of curiosity and wonder, promoting emotional resilience and joy (Cabana et al., 2024). On the other hand, the absence or degradation of these natural environments can exacerbate negative mental health outcomes. Awareness of environmental crises, such as climate change and biodiversity loss, may lead to feelings of anxiety, grief, or helplessness, particularly among children learning about these issues in educational settings. However, these emotional responses can often be mitigated by incorporating actionable solutions and collaborative experiences that emphasize positive engagement with nature.

#### 3.3.5 The relationship between blue and green spaces and children’s physical health

Urban blue and green spaces play a crucial role in promoting children’s physical health by encouraging higher levels of physical activity, supporting weight management, and fostering healthy lifestyle habits (Vidal and Castro Seixas, 2022; Russo et al., 2024). Access to well-maintained parks and playgrounds has been consistently linked to increased physical activity, both of which are critical for reducing sedentary behavior and improving overall fitness (Molina-García et al., 2021). Additionally, children living in areas with higher greenness, as measured by Normalized Difference Vegetation Index (NDVI), are less likely to experience obesity due to greater opportunities for outdoor activities and reduced screen time (Bellisario et al., 2022). Blue spaces, such as rivers and coastal areas, further contribute to physical health by offering diverse activity options (Crooks et al., 2022). However, the quality and accessibility of these spaces, including cleanliness, safety, and the presence of recreational facilities, significantly influence their usage and benefits (Jiang et al., 2023). Urbanized areas often face challenges such as limited green space availability, which can reduce physical activity levels and increase obesity risk.

### 3.4 Analysis of research frontiers

**Figure 5:**
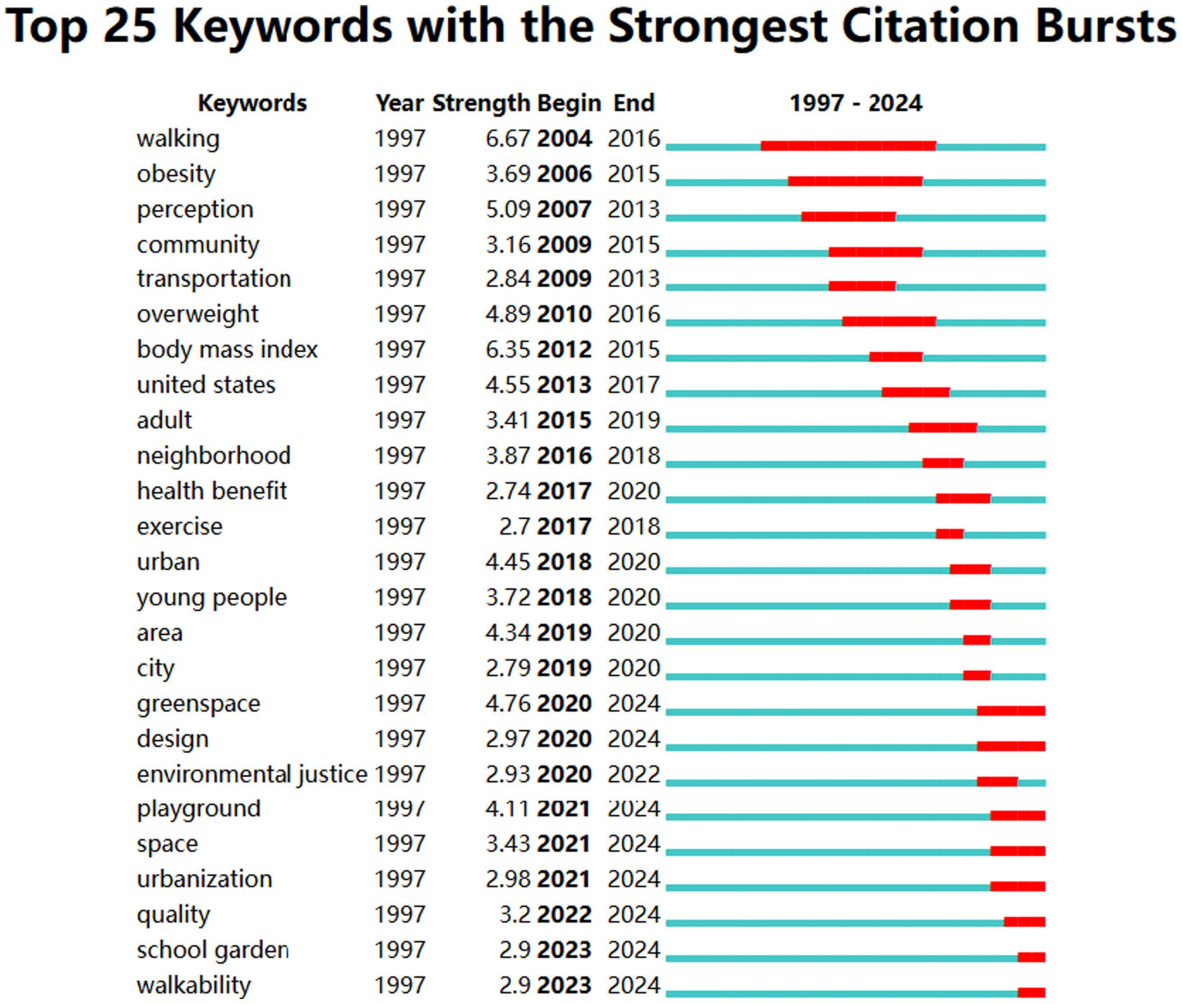
Top 25 keywords with the strongest citation bursts.

The keyword burst analysis reveals that the research field on the impact of urban blue and green spaces on children’s health can be divided into three distinct phases (Figure 5). The early phase (2000–2015) focused primarily on individual health and physical activity. The transition phase (2015–2020) saw an expansion toward exploring the broader relationship between urban environments and health benefits. Finally, the recent phase (2020–2024) shifted attention to sustainability, quality, and walkability.

During the early phase (2005–2015), the primary focus was on individual health outcomes, particularly related to physical activity and weight management. Keywords such as “walking,” “obesity,” “body mass index,” “perception,” “community,” and “transportation” were prominent. These studies explored how urban spaces influenced physical health indicators like obesity (Martenies et al., 2024) and physical activity (Huang et al., 2024), emphasizing the role of urban design and transportation in promoting healthy behaviors. This phase laid the foundation for understanding the connection between urban environments and health outcomes, with a stronger emphasis on physical rather than mental health.

From 2015 to 2020, the research shifted from individual health to a broader exploration of the urban environment’s impact on health. Keywords such as “health benefit,” “exercise,” “urban,” “neighborhood,” and “area” became significant. This phase marked the beginning of an interest in the interplay between urban neighborhoods, green spaces, and health outcomes. Researchers began to explore the specific mechanisms (Asah et al., 2017) through which urban environments influence health, such as access to exercise opportunities (Klomsten Andersen et al., 2024) and neighborhood design (Bao et al., 2023). Mental health benefits began to emerge as a critical focus during this period.

The most recent phase (2020–2024) demonstrates a significant shift toward sustainability, quality, and walkability. Keywords such as “greenspace,” “design,” “environmental justice,” “playground,” “urbanization,” “quality,” and “walkability” highlight an emphasis on equitable access to urban green spaces (Liao and Furuya, 2024), sustainable urbanization, and the psychological impacts of specific urban interventions. Notably, terms like “school garden” (Kwok et al., 2021) and “playground” (Molina-García et al., 2021) suggest a growing interest in how specific environments, such as schools and pedestrian-friendly spaces, can promote mental health and well-being among children (Din et al., 2023).

### 3.5 Co-citation analysis of references

**Figure 6:**
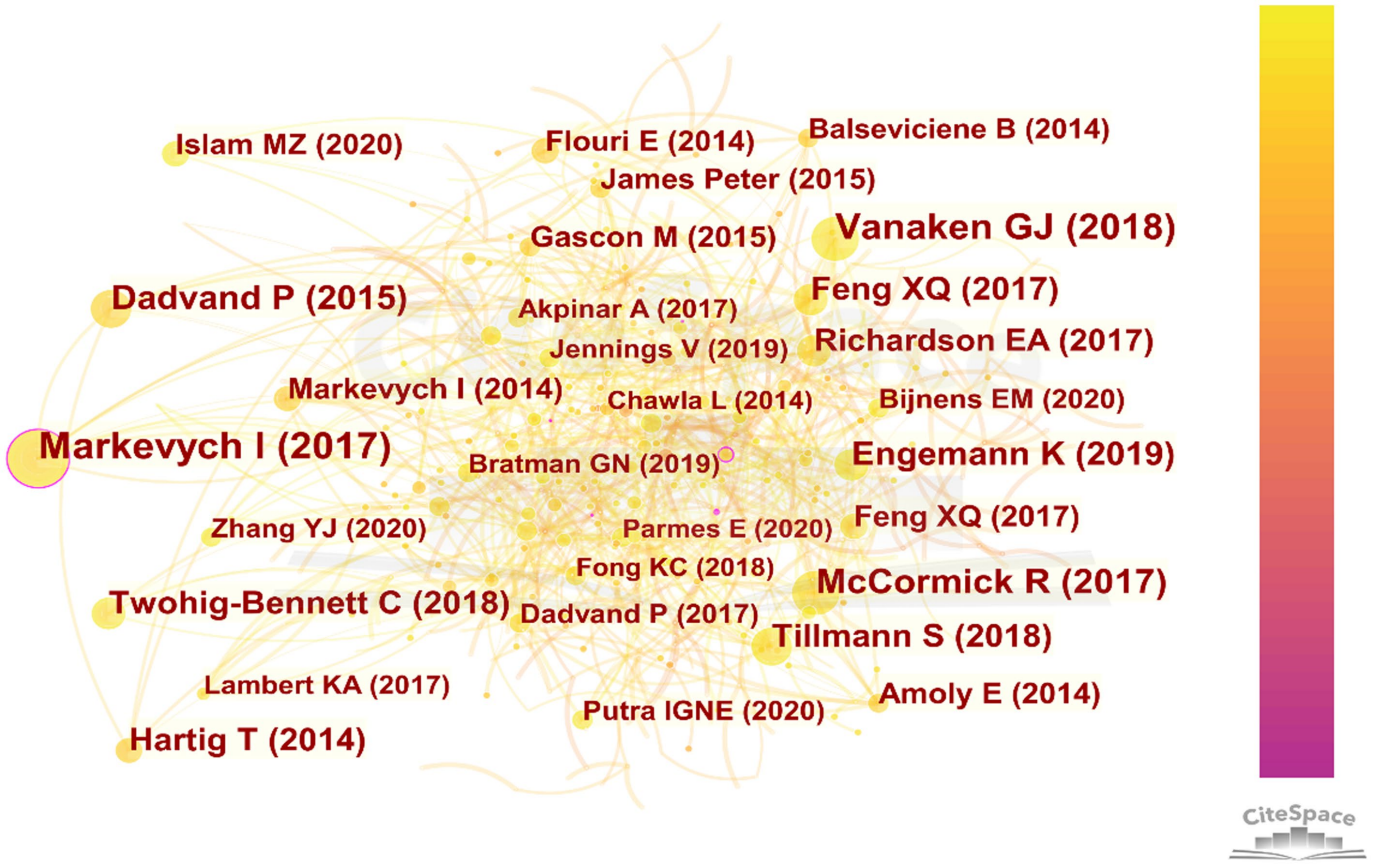
Visualization of the co-citation network of references.

Figure 6 shows the visualization of the co-citation network of references. The 10 papers in Table 7, published between 2014 and 2019, reflect the growing and evolving interest in the relationship between urban blue and green spaces and children’s health. Most were published during 2014–2015 (6 out of 10), marking a surge in foundational research, while later publications (2017–2019) shifted toward broader theoretical frameworks, systematic reviews, and longitudinal studies. These papers were published in a variety of high-impact and specialized journals, including PNAS, Environmental Research, Environment International, and the International Journal of Environmental Research and Public Health, highlighting the interdisciplinary nature of the field. Overall, the earlier studies focused on specific relationships, such as cognitive development and behavior, while recent works provide comprehensive evidence and methodological insights, advancing the field toward more systematic and policy-relevant research. Among them, three notable studies specifically examine how these spaces influence children’s behavior. These studies shed light on a critical yet underexplored dimension of this research field, providing valuable insights into the behavioral benefits of blue and green spaces.

**Table 7:** Top 10 references with the strongest citation bursts.

No.	Freq.	Burst	Author	Year	Source	Title
1	29	11.21	Dadvand P	2015	PNAS	Green spaces and cognitive development in primary schoolchildren
2	44	10.31	Markevych I	2017	Environmental Research	Exploring pathways linking greenspace to health: Theoretical and methodological guidance
3	23	10.14	Hartig T	2014	Annual Review of Public Health	Nature and Health
4	19	8.36	Amoly E	2014	Environmental Health Perspectives	Green and Blue Spaces and Behavioral Development in Barcelona Schoolchildren: The BREATHE Project
5	18	7.92	Markevych I	2014	Environment International	Access to urban green spaces and behavioural problems in children: Results from the GINIplus and LISAplus studies
6	19	7.31	Gascon M	2015	International Journal of Environmental Research and Public Health	Mental Health Benefits of Long-Term Exposure to Residential Green and Blue Spaces: A Systematic Review
7	31	7.08	McCormick R	2017	Journal of Pediatric Nursing	Does Access to Green Space Impact the Mental Well-being of Children: A Systematic Review
8	27	6.62	Engemann K	2019	PNAS	Residential green space in childhood is associated with lower risk of psychiatric disorders from adolescence into adulthood
9	15	6.59	Balseviciene B	2014	International Journal of Environmental Research and Public Health	Impact of Residential Greenness on Preschool Children’s Emotional and Behavioral Problems
10	24	5.24	Tillmann S	2018	Journal of Epidemiology and Community Health	Mental health benefits of interactions with nature in children and teenagers: a systematic review

Urban blue and green spaces significantly influence children’s behavior and mental health through multiple mechanisms, as revealed by three complementary studies. Regular exposure to green and blue spaces, such as parks and beaches, has been shown to reduce stress, improve attention, and decrease behavioral issues. Increased interaction with green and blue spaces correlated with reductions in emotional problems, peer relationship issues, and ADHD symptoms (Amoly et al., 2014). Similarly, research in Munich found that children living more than 500 meters from urban green spaces had a higher risk of hyperactivity and attention problems, with boys particularly affected, highlighting the importance of proximity (Markevych et al., 2014). In Lithuania, while higher residential greenness benefited children from lower-education households, it was paradoxically associated with increased behavioral problems among children from higher-education households (Balseviciene et al., 2014).

These effects are mediated by mechanisms such as psychological restoration, enhanced physical activity, reduced exposure to pollution and noise, and opportunities for social interaction. Immediate green space proximity (e.g., within 100 or 300 meters) has been associated with direct psychological benefits like stress reduction, while larger green spaces tend to support physical activity and social behaviors. Collectively, these findings emphasize the need for equitable access to well-maintained blue and green spaces in urban areas to foster children’s healthy behavioral and psychological development.

## 4 Discussion

**Figure 7:**
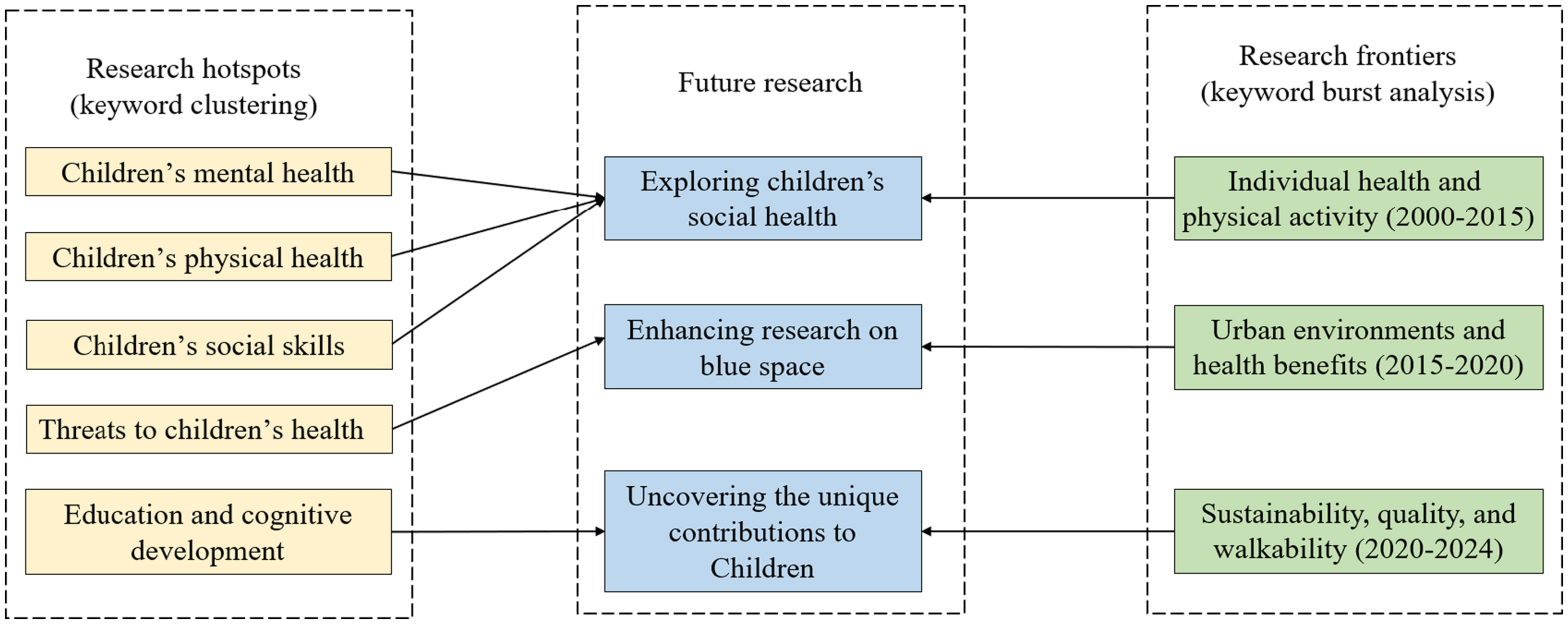
Existing research and opportunities for future research.

Based on the results of keyword clustering and keyword burst analysis (as shown in Figure 7), this study suggests three major directions for future research. These directions not only reflect the evolving focus of existing literature but also point toward emerging gaps that warrant further exploration. First, future studies should prioritize enhancing the role of blue spaces in urban research, recognizing their potential health and developmental benefits for children. Second, more attention should be paid to exploring the social health impacts of both blue and green spaces on children. Third, it is essential to uncover the unique contributions of blue and green spaces to children.

### 4.1 Enhancing the blue spaces in urban research

Emphasis on blue spaces is crucial for understanding the relationship between urban environments and children’s health. While much of the existing research has focused predominantly on the health benefits of green spaces, the unique contributions of blue spaces, such as rivers, lakes, and oceans, remain underexplored (Rouse and Wishart, 2024). Unlike green spaces, blue spaces offer unique, water-related opportunities that substantially differ in their mechanisms and magnitude of health impact. Specifically, blue spaces provide exclusive opportunities for water-based physical activities like swimming, boating, kayaking, and other forms of aquatic recreation, which are generally unavailable in typical green parks or urban gardens (White et al., 2020). These activities not only promote physical fitness but also encourage skill development among children (Foley and Kistemann, 2015). Moreover, blue spaces have been shown to offer enhanced psychological benefits distinct from green spaces, such as stronger stress reduction effects due to the calming sensory experience of water, often termed the “blue effect” (Gascon et al., 2017). The rhythmic sounds and visuals associated with water environments can uniquely contribute to relaxation, emotional restoration, and mental well-being in children, potentially more intensively than green spaces alone (Nutsford et al., 2016).

Another unique attribute of blue spaces is their ecological and biodiversity value. These areas typically support diverse aquatic and semiaquatic ecosystems, offering richer biodiversity experiences compared to typical urban green spaces (Völker and Kistemann, 2011). Direct exposure to diverse wildlife and ecosystems can significantly enhance children’s environmental awareness, emotional connections with nature, and educational experiences (Keniger et al., 2013). Moreover, existing research rarely addresses the potential synergistic effects of combined green and blue spaces. Urban areas integrating both types of spaces—such as parks adjacent to lakes or riverside greenways—may amplify health benefits through diversified recreational opportunities, varied sensory experiences, and increased social interactions among children (Bell et al., 2018). Such integrated environments could offer enhanced restorative effects and encourage greater levels of physical and social engagement compared to isolated green or blue spaces (Britton et al., 2020).

### 4.2 Exploring the social health impacts of blue and green spaces on children

While the relationship between blue and green spaces and children’s physical and mental health has been well-documented (Wu et al., 2021), their impact on children’s social health remains underexplored. Social health, encompassing aspects such as social skills, peer interactions, and a sense of community, is a critical component of overall well-being, particularly during childhood. Urban blue and green spaces can serve as valuable settings for promoting social health by encouraging group activities, fostering social interactions, and providing inclusive environments where children can develop teamwork and communication skills. Research on how blue and green spaces facilitate social interactions is still limited, but the potential benefits are evident. For instance, playgrounds in green spaces or accessible waterfront areas can create opportunities for children to engage in cooperative play, build friendships, and strengthen peer relationships (George et al., 2024). Additionally, well-designed natural spaces can reduce social inequalities by offering inclusive spaces that accommodate children from diverse socioeconomic backgrounds. Future studies should investigate the mechanisms through which blue and green spaces influence social health, considering factors such as design, accessibility, and cultural context.

### 4.3 Uncovering the unique contributions of blue and green spaces to children

While the physical, mental, and social health benefits of blue and green spaces are widely recognized across all age groups, including children, middle-aged adults, and the elderly, it is essential to explore the unique impacts of these spaces on children. As individuals whose cognitive and emotional capacities are still developing, children experience blue and green spaces differently. These natural environments serve as vital platforms for exploration, learning, and cognitive development, offering opportunities for children to engage with nature, stimulate curiosity, and foster creativity. Blue and green spaces provide children with a unique setting to embrace and perceive nature, contributing significantly to their cognitive and emotional growth. Activities in such environments, such as interacting with plants, observing wildlife, or simply playing in green areas, enhance both intellectual (IQ) and emotional intelligence (EQ). Through these interactions, children not only develop critical thinking but also cultivate empathy, patience, and social awareness. Moreover, as children learn about the ecosystem, plants, and natural processes, their understanding of the environment deepens, fostering a lifelong connection to nature and a sense of responsibility toward preserving it. Future research should focus on the developmental aspects of blue and green spaces for children, investigating how these spaces contribute to specific cognitive milestones and emotional well-being.

### 4.4 Policy implications

Our study offers several practical implications for policymakers and urban planners seeking to promote children’s health through the provision of green and blue spaces. First, the findings highlight the importance of incorporating a variety of multifunctional natural spaces, including parks, riversides, school gardens, and green schoolyards, into urban planning. These spaces should not only be available but also equitably distributed to ensure that children from all socioeconomic backgrounds can benefit from their physical, mental, and social health advantages. In addition, green and blue spaces should be designed with children’s needs in mind, balancing safety, playability, and ecological richness. By fostering opportunities for unstructured play, physical activity, exploration, and quiet reflection, these environments can promote holistic development. Second, the study highlights the importance of ensuring environmental quality in urban natural spaces. Policymakers should strengthen environmental monitoring and maintenance to address potential threats such as soil contamination, water pollution, and exposure to hazardous materials. Clean, safe, and accessible environments are essential for realizing the full health potential of urban nature. Third, integrating nature-based education and mental health programs into schools and community services can further amplify the benefits of green and blue spaces. Initiatives such as outdoor learning environments, school gardening programs, and nature therapy can foster cognitive development, reduce stress, and cultivate environmental awareness and stewardship from an early age.

## 5 Conclusion

Our study provides a comprehensive bibliometric analysis of research on the impact of urban blue and green spaces on children’s health. This field demonstrates an interdisciplinary nature, encompassing public health, environmental science, and urban studies. Collaborative networks among countries, authors, and institutions highlight the global and interconnected nature of this research, with the United States, Australia, and European countries acting as key hubs of academic activity. The findings reveal significant growth in academic interest over three distinct phases: an early focus on individual health and physical activity (2000–2015), a transition to broader exploration of urban environments and health benefits (2015–2020), and a recent emphasis on sustainability, quality, and walkability (2020–2024). Keyword clustering analysis further identifies five key research hotspots: physical health benefits, mental health impacts, social skill development, educational and cognitive outcomes, and environmental threats posed by blue and green spaces. While the benefits of green spaces have been extensively studied, blue spaces remain underrepresented despite their potential to contribute uniquely to children’s health. Additionally, we further identify future research trends in the study of urban blue and green spaces and their impact on children’s health. Our study provides a foundational understanding of this field and serves as a reference for future scholars conducting related research.

## Data Availability

The raw data supporting the conclusions of this article will be made available by the authors, without undue reservation.
